# Laboratory demonstration of a prozone-like effect in HRP2-detecting malaria rapid diagnostic tests: implications for clinical management

**DOI:** 10.1186/1475-2875-10-286

**Published:** 2011-09-29

**Authors:** Jennifer Luchavez, Joanne Baker, Sheila Alcantara, Vicente Belizario, Qin Cheng, James S McCarthy, David Bell

**Affiliations:** 1 Department of Parasitology, Research Institute for Tropical Medicine, Alabang, Muntinlupa City, The Philippines; 2 Drug Resistance and Diagnostics, Australian Army Malaria Institute, Brisbane, Australia; 3 Department of Developmental Biology, The University of Texas Southwestern Medical Center, Dallas, TX, USA; formerly Department of Parasitology, Research Institute for Tropical Medicine, Alabang, Muntinlupa City, Philippines; 4 Queensland Institute of Medical Research, University of Queensland, Brisbane, Australia; 5 Foundation for Innovative New Diagnostics (FIND), Geneva, Switzerland; formerly World Health Organization - Regional Office for the Western Pacific, Manila, The Philippines

## Abstract

**Background:**

Malaria rapid diagnostic tests (RDTs) are now widely used for prompt on-site diagnosis in remote endemic areas where reliable microscopy is absent. Aberrant results, whereby negative test results occur at high parasite densities, have been variously reported for over a decade and have led to questions regarding the reliability of the tests in clinical use.

**Methods:**

In the first trial, serial dilutions of recombinant HRP2 antigen were tested on an HRP2-detectiing RDT. In a second trial, serial dilutions of culture-derived *Plasmodium falciparum *parasites were tested against three HRP2-detecting RDTs.

**Results:**

A prozone-like effect occurred in RDTs at a high concentration of the target antigen, histidine-rich protein-2 (above 15,000 ng/ml), a level that corresponds to more than 312000 parasites per μL. Similar results were noted on three RDT products using dilutions of cultured parasites up to a parasite density of 25%. While reduced line intensity was observed, no false negative results occurred.

**Conclusions:**

These results suggest that false-negative malaria RDT results will rarely occur due to a prozone-like effect in high-density infections, and other causes are more likely. However, RDT line intensity is poorly indicative of parasite density in high-density infections and RDTs should, therefore, not be considered quantitative. Immediate management of suspected severe malaria should rely on clinical assessment or microscopy. Evaluation against high concentrations of antigen should be considered in malaria RDT product development and lot-release testing, to ensure that very weak or false negative results will not occur at antigen concentrations that might be seen clinically.

## Background

Rapid and accurate diagnosis is key to effective treatment and management of malaria [1]. The wide use of lateral flow rapid diagnostic tests (RDT) is essential to achieve this, as microscopy is impractical in many areas. The safety and credibility of many diagnostic programmes relies therefore on the accuracy of RDTs. While some RDTs have long proven effective in field and laboratory studies and more recently in wide-scale routine use [2-8], false negative results, in particular, have potential for harming patient health and damaging the credibility of malaria control programmes. Failure to detect a case of malaria parasitaemia could lead a clinician to withhold potentially life-saving anti-malarial therapy that would have been dispensed if non-specific symptom-based diagnosis had been used.

As is the case for all lateral flow immunochromatographic diagnostic tests, malaria RDTs have limitations due to their reliance on specific antigen-antibody interactions that can be subject to a range of interfering factors, and due to other device-related failures and operator errors. While these devices detect parasite antigen in host blood rather than actual malaria parasites, a semi-quantitative relationship between parasitaemia and intensity of the positive result has been reported in some studies [9-12]. However, many factors may affect the relationship between parasite density and antigen concentration, and detection of antigen by the lateral flow device. In addition to variable sensitivity at low parasite density [4,9,13-15], there have long been reports of unexplained negative results at high parasite density [11,16-20]. Possible explanations of these reports have included gene deletions [21], variation in antigen structure [22,23], or a prozone-like effect [24-26].

The prozone phenomenon (high dose hook effect) is a well-recognized phenomenon in a range of immunologic assays depending on antigen-antibody interactions, including rapid antibody-detecting immunological diagnostic tests. They occur when high antibody concentration saturates antigen and prevents lattice formation and precipitation [27-31]. Similar prozone-like effects are observed with antigen assays for a number of applications [32-35], and observed in certain malaria tests [24].

A prozone-like effect occurring with malaria RDTs in patients who have very high parasite density could lead to inappropriate withholding of antimalarial treatment to patients who require urgent therapy, with an isolated case recently being reported [24]. This paper reports an investigation of the potential of a prozone-like effect to cause false negative RDT results. The effect of adding varying amounts of recombinant HRP-2 (rHRP-2) to a commercially-available malaria RDT designed to detect this common target antigen was observed, and the study repeated using high-density culture-derived *Plasmodium falciparum *parasites.

## Methods

The study was performed in two parts; Trial 1 using recombinant HRP2 in the Research Institute for Tropical Medicine, the Philippines, in 2004, and Trial 2 using cultured *P. falciparum *in the Army Malaria Institute (AMI), Australia in 2009.

### Trial 1

#### Preparation of antigen dilutions

To assess the effect on the RDT of very high antigen concentration, recombinant HRP-2 (National Bioproducts Institute [Dr Martin Bubb], Pinetown, South Africa) was serially diluted from an initial concentration of 1.5 mg/mL with parasite-negative type "O" blood down to 1:100,000, with resultant antigen concentrations ranging from 1,500,000 ng/mL to 15 ng/mL.

To determine equivalent parasite densities to the recombinant antigen used, the value of 9.6 ng/mL at 200 parasite/μL is used, based on the median value of 79 infected patients diluted to 200 parasites/μL reported for the evaluation panel used in the first round of the WHO product testing programme [36,37]. An ELISA method had been used to determine these concentrations, as detailed in version 1 of the Methods Manual for the WHO product testing programme [38]. This indicates an equivalent parasite density range of 31 × 10^6 ^(biologically implausible) to 313 parasites/μL (Table 1).

**Table 1:** Recombinant HRP2 concentrations used in Trial 1, and equivalent parasite densities derived from the median HRP2 concentration of dilutions of 200 parasite/μL of the WHO global specimen bank

HRP2 concentration (ng/mL)	Line intensity (densitometer)	Mean line intensity (3 readers)	Calculated parasite density equivalent (parasite/μL)
15	1.8	1.3	313
150	7.2	3.0	3,125
1,500	16.9	4.0	31,250
15,000	17.9	4.0	312,500
150,000	13.0	3.7	3,125,000
300,000	10.8	3.0	6,250,000
500,000	5.2	2.3	10,416,667
750,000	4.4	2.0	15,625,000
1,000,000	2.4	2.0	20,833,333
1,500,000	1.5	1.0	31,250,000

#### RDTs

The serially diluted antigen preparations were tested on a HRP-2 detecting RDT, Paracheck *Pf *(Orchid Biomedical Systems, India; Lot Number 31083A; Expiry 04/2005), which had been stored at 4°C. The RDT was prepared according to the manufacturer's instructions, with micropipettes used to ensure exactly 5 μL of blood from each antigen dilution was pipetted onto the sample well. Six drops of buffer were added to the buffer well immediately. After 15 minutes, the results were read visually by three experienced technicians, comparing the colour intensity of the test band against a standard RDT rating chart developed previously by WHO and RITM, rated from 0 (no band or negative), 1 (very faint) to 4 (strong line) [39]. The average of the three blinded readings was recorded for each test. The intensity of each test band was measured in parallel using a densitometer designed for lateral flow tests ('Videolab', Protea-Synteco, Moscow, Russia).

### Trial 2

#### Cultured *P. falciparum *parasites

Five culture-adapted *P. falciparum *lines originating from different geographic areas (GA3, FCR3, SJ44, S55 and 7G8) were cultured *in vitro*, as previously described [22]. Parasite cultures were repeatedly synchronized using 5% sorbitol [40], and harvested when parasitaemia reached ≥10%, with a majority of parasites at ring stage. The percentage parasitaemia at which parasite lines SJ44, S55, FCR3, 7G8 and GA3 were harvested were 10%, 16%, 16%, 16% and 17%, respectively. A separate culture of 7G8 was harvested at a parasitaemia of 25%.

#### Dilutions of the parasites

An aliquot of each harvested parasite (3% haematocrit) was serially diluted 10 fold five times using normal red blood cells at 3% haematocrit. An aliquot of the undiluted parasite and each dilution was tested on RDTs. To imitate the conditions of patient blood, another aliquot of the harvested parasites was first concentrated to 50% haematocrit, then serially diluted 10 fold five times using normal human blood at 50% haematocrit. Aliquots of the undiluted blood and each of the dilutions were also tested on RDTs. It should be noted that an undiluted culture with 10% parasitaemia at 3% and 50% haematocrit would have ~30000 and ~500000 parasites/μL, respectively.

#### Performing rapid diagnostic tests

Three RDTs for malaria were used to test serial dilutions of the cultured parasites: ICT Malaria Combo Cassette Test (ML02, ICT Diagnostics, South Africa), First Response Malaria Antigen pLDH/PfHRP2 Combo (Cat. No. 116FRC30, Premier Medical Corporation, India) and SD Malaria Antigen *P.f *(05FK50, Standard Diagnostics, Korea). All three tests detect *P. falciparum *using antibodies against PfHRP2. The tests were performed according to the manufacturer's instructions. The results were read by two independent readers and recorded as levels 0-4 based on the test band intensity following the same WHO colour reference chart [39].

#### Ethics approval

The recombinant antigen challenge study was approved by the Ethics Review Board of the Research Institute for Tropical Medicine, Philippines. Trial 2, using cultured parasites, was approved under the Australian Defence Human Research Ethics Committee approval number 377-05.

## Results

### Trial 1

**Figure 1:**
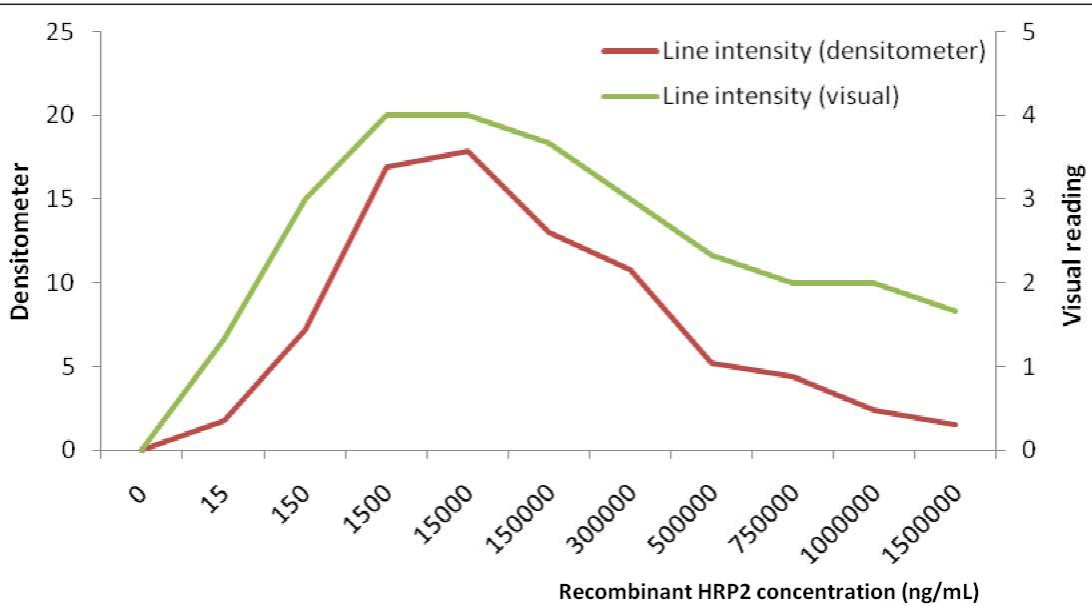
**RDT line intensity measured by densitometer and visual rating chart at various dilutions of recombinant HRP2**. Note that x-axis scale is arbitrary.

Both visual and densitometer readings showed an overall non-linear relationship with HRP2 concentration and calculated equivalent parasite density (Figure 1). The mean visual intensity of the test band increased rapidly from 1 (faint) at 1.5 mg/mL to 4 (strong band) at 1,500 mg/mL of HRP2, but dropped to only 1.3 as the antigen concentration reached maximum (1,500,000 ng/mL or 1.5 mg/mL). Densitometer readings showed a trend similar to the visual readings, with the greatest colour intensity of the test band using either method observed between rHRP-2 concentrations of 1,500 - 150,000 ng/mL (Figure 1). While the intensity of the test band markedly decreased below the peak intensity at antigen concentrations above this level, the bands remained visible.

Assuming that the relationship between parasite density and antigen concentration remains constant across parasite densities, the parasite density equivalent to the HRP2 concentration at which line intensity declined (15,000 ng/mL) corresponds to >312,500 parasite/μL.

### Trial 2

Irrespective of the starting parasite density (measured as percent of red cells), testing of all undiluted parasite samples returned a positive result in all 3 RDTs tested. At parasite densities of 10-16%, the undiluted sample gave the highest test band intensity, with some diminution at lower densities. At parasite densities exceeding 17-25% however, the test bands in the undiluted samples was thinner and qualitatively of reduced intensity compared to the results seen at the first dilution, an observation that remained constant irrespective of the haematocrit of the sample (Table 2), i.e. the peak band intensity was observed in the 1^st ^dilution. The band intensity decreased after the 3^rd ^dilution.

**Table 2:** Detection results of dilutions of cultured parasites on three brands of RDTs in Trial 2

Line & Origin	Parasitaemia %	3% HCT			50% HCT		
		
		ICT	SD	FR	ICT	SD	FR
SJ44	10	4	4	4	4	4	4
	
Solomon Islands	1	4	4	4	4	4	4
	
	0.1	4	3	2	3	3	1
	
	0.01	1	1	0	0	1	0
	
	0.001	1	0	0	0	0	0
	
	0.0001	1	0	0	0	0	0

FCR3	16	ND	4	4	ND	4	4
	
Gambia	1.6	ND	4	4	ND	4	4
	
	0.16	ND	4	4	ND	4	4
	
	0.016	ND	4	3	ND	3	2
	
	0.0016	ND	2	1	ND	1	0
	
	0.00016	ND	0	0	ND	0	0

GA3	17	4	4	4 thin	4	4	4 thin
	
Thailand	1.7	4	4	4	4	4	4
	
	0.17	4	4	4	4	4	4
	
	0.017	3	3	3	2	3	3
	
	0.0017	1	1	1	1	1	1
	
	0.00017	0	0	0	0	0	0

S55	16	ND	4	4	ND	4	4
	
Solomon Islands	1.6	ND	4	4	ND	4	4
	
	0.16	ND	4	4	ND	4	4
	
	0.016	ND	2	2	ND	2	2
	
	0.0016	ND	1	0	ND	0	0
	
	0.00016	ND	0	0	ND	0	0

7G8	16	ND	4	4	ND	4	4
	
Brazil	1.6	ND	4	4	ND	4	4
	
	0.16	ND	4	4	ND	3	4
	
	0.016	ND	2	2	ND	2	1
	
	0.0016	ND	0	0	ND	0	0
	
	0.00016	ND	0	0	ND	0	0

7G8	25	4 thin	3	4 thin	4 thin	4 thin	4 thin
	
Brazil	2.5	4	4	4	4	4	4
	
	0.25	4	4	4	4	4	4
	
	0.025	4	4	4	4	2	3
	
	0.0025	1	1	2	2	1	1
	
	0.00025	1	0	0	0	0	0

Note: ICT- ICT Malaria combo Cassette Test, ICT Diagnostics, South Africa; FR- First Response Malaria Antigen pLDH/HRP2 Combo, Premier Medical Corporation, Limited, India; SD- SD Malaria Antigen *P.f*, Standard Diagnostics, Korea.

## Discussion

A false negative RDT result, or a failure to appreciate the severity of *P. falciparum *infection, may have life-threatening consequences for a sick individual denied appropriate treatment. In this study, a marked reduction in intensity of RDT test lines was observed at high antigen concentration, consistent with a prozone-like effect. Importantly however, the test band did not totally disappear up to the highest antigen level or parasitaemia tested (1.5 mg/mL and ~500,000 parasites/μL respectively). The optimum range of HRP-2 concentrations in terms of line intensity was ~1,500 to 15,000 ng/mL (equivalent to ~31,250 and ~312,500 parasites/μL respectively). The results confirm a roughly linear trend in the relationship between line intensity and antigen concentration below that level, thus supporting previous observations of the semi-quantitative properties of RDTs at lower parasite densities, a relationship that disappears at higher densities. At antigen concentration above this range, i.e. 150,000 ng/mL, where the test band intensity in Trial 1 started to plateau, the expected parasite density (Table 1) would be around 3.1 million parasites per μL of blood, a biologically implausible level. However, antigen concentration varies widely with peripheral parasite density. The range of antigen concentration in the 200 parasite/μL WHO panel is very wide, ranging from 73.7 ng/mL to 0.8 ng/mL after outliers were excluded [36]. Taking the higher value, the antigen concentration at which test line declined may occasionally occur at with a parasite density as low as 170,000 parasites/μL, which is within the range seen clinically in some cases of severe malaria. Therefore, while the line did not disappear at this level providing a false-negative result, a faint line occurring could mislead a clinician to overlooking hyperparasitaemia.

The results of Trial 2 using cultured parasites are broadly consistent with the findings using recombinant protein: at maximum parasite densities ranging from 10% at 50% haematocrit to 25% at 50% haematocrit (~500,000 to 1.25 million parasites/μL), the intensity of lines did not diminish with any of the six different laboratory strains or with any of the three different RDTs used. However, some 'thinning' of lines indicated that a prozone-like effect may have been beginning to impact the results at higher parasite densities.

Gillet *et al *reported a prozone-like effect in a number of HRP2-detecting tests at parasitaemias ranging from 5.5% to 35%. These consisted of reduced line intensities at maximum concentration that resolved after dilution. Although some line intensities were reported as faint at high parasite densities, in only a single case (1 of 16 RDT brands at 11.5%) did this result in a test reading negative [24].

The mechanism underlying the prozone-like effect is not well defined for antigen-detecting tests. One plausible explanation is that excess antigen remains unbound after saturation of the signal monoclonal antibody (Mab), and passes up the strip to bind the capture Mab on the RDT test line. If this target antigen reaches the test line in sufficient quantities, it could saturate the capture antibody, thereby preventing the signal Mab-antigen complex from binding and accumulating into a visible band. The likelihood of a false negative result at high concentration would therefore depend not only on target antigen concentration (which may vary widely with parasite density), but also on whether too little capture antibody is present in the test line of the RDT.

The demonstration here of a weak prozone-like effect at a very high antigen concentrations and parasite densities in HRP2-detecting RDTs is consistent with previous observations. Importantly no false negative results were observed, and indeed previous observations also suggest that this is rare. However, a combined effect whereby the non-linear relationship between antigen concentration and parasite density, and variation in the quality of RDT manufacture, could combine to result in a false negative test is conceivable, and in this circumstance could lead a clinician to miss a malaria diagnosis, as demonstrated in the case report quoted earlier[24].

## Conclusions

These results and those previously published have a number of implications. Firstly, RDTs should not be viewed as quantitative tests. The plateau and fading effect may mislead the clinician to underestimate a potentially life-threatening hyperparasitaemia. Secondly, as recommended by WHO [1], patients with clinically severe illnesses in malaria-endemic areas should receive immediate treatment with anti-malarial drugs, and appropriate antibiotics, and referred for definitive diagnosis. Thirdly, the quality of manufacture of RDTs is likely to influence the likelihood of a prozone-like phenomenon resulting in false negative test results. Thus, further RDT development, and lot-release testing of RDTs, could usefully take this into account and include evaluation against high concentrations of antigen to ensure that false negative results will not occur at antigen concentrations that might be seen clinically.

## Competing interests

The authors declare that they have no competing interests.

## Authors' contributions

JL, SA, VB performed and reported Trial 1. JB, QC, JM performed and reported Trial 2. DB coordinated the project and led manuscript writing. All authors contributed to and approved the manuscript.
